# Rethinking intrinsic capacity in the age of artificial intelligence: a new materialist gerontology perspective

**DOI:** 10.1093/geroni/igag098

**Published:** 2026-11-03

**Authors:** Bo Liang, Gong Chen

**Affiliations:** Institute of Population Research, Peking University, Beijing, China; Institute of Population Research, Peking University, Beijing, China

**Keywords:** Algorithmic assessment, Distributed capacity, Healthy aging, Sociotechnical systems

Artificial intelligence (AI) systems are increasingly being integrated into intrinsic capacity (IC) assessment, frailty prediction, and care decision-making in later life. These developments align with the World Health Organization (WHO) IC framework, which defines IC as the composite of an individual’s physical and mental capacities and distinguishes it from functional ability, which also depends on environmental conditions (WHO, 2015). Most debates about AI in aging have focused on whether these systems are accurate, acceptable, efficient, or biased. These questions are important, but they leave a deeper issue under-examined: AI-enabled IC assessment is not only a measurement practice; it is also a capacity-making practice. Here, AI systems refer to the broader sociotechnical infrastructures, including algorithms, digital platforms, sensors, and data architectures, that support IC assessment, whereas AI tools refer to specific applications operating within these systems.

Situated within the broader tradition of critical gerontology, new materialist gerontology extends earlier critiques of aging by emphasizing that aging is produced not only through social institutions but also through relations among bodies, technologies, infrastructures, and data (Höppner & Urban, 2018). “Material” refers not simply to physical objects but to these heterogeneous assemblages through which aging capacities emerge. This perspective shifts attention from AI as an external tool to AI as part of the sociotechnical arrangements that enact, measure, classify, and govern IC (Barad, 2007).

From this relational perspective, AI assessment systems shape how capacity becomes visible and actionable through three interconnected processes: measurement, classification, and governance (Barad, 2007; Höppner & Urban, 2018). First, at the measurement level, AI systems transform lived bodily experience into measurable signals. Digital IC tools and wearable sensors do not capture the body in its entirety; they select and standardize particular aspects of cognition, mobility, vitality, or sensory function. The WHO ICOPE Monitor App, for example, assesses IC through standardized tasks and prompts embedded in a digital interface. Studies of ICOPE implementation show variation between self-administered and clinician-administered assessments, particularly in cognitive and sensory domains, suggesting that outcomes depend not only on individual condition but also on how assessment is configured (Gonzalez-Bautista et al., 2023; Tavassoli et al., 2022). Similarly, wearable mobility sensors generate indicators such as gait speed or stride variability that are often treated as objective markers of frailty, yet these measures depend on sensor placement, sampling conditions, and algorithmic processing. Measurement therefore produces a technologically mediated version of capacity.

Second, at the classification level, measured signals are transformed into categories such as “healthy,” “pre-frail,” or “frail.” Machine-learning models may demonstrate predictive performance, but the boundaries through which frailty is defined are shaped by training data, feature selection, and validation criteria rather than by a universally stable biological line. Evidence from IC screening studies shows wide variation in estimates of IC loss across settings, suggesting that different assessment systems may classify different groups of older adults as “frail” (de Oliveira et al., 2023). These classifications also have equity implications. Algorithmic systems often underrepresent marginalized older adults, including minoritized and low-income groups, in training datasets, increasing the risk that their capacities and vulnerabilities are misclassified (Chu et al., 2023). The issue is therefore not only whether AI classifications are accurate, but how they define whose needs become recognizable.

Third, at the governance level, classifications become embedded in institutional decision-making. Frailty scores and risk alerts may shape service eligibility, care planning, and mobility restrictions. In long-term care settings, algorithmic monitoring and risk scoring can gradually displace relational forms of professional judgment (Berridge & Grigorovich, 2022). When care workers act on algorithmic outputs that they cannot fully interpret, accountability becomes diffuse. Recent work on AI in long-term care shows that responsibility for adverse outcomes is often unclear when decisions emerge from interactions among software systems, institutional protocols, and care infrastructures (Lukkien et al., 2023). Once algorithmic classifications enter care planning, IC is no longer only a clinical assessment construct; it becomes part of institutional decisions about eligibility, monitoring, and responsibility.

Taken together, these processes suggest that IC should not be understood as a stable reserve located solely within the individual body. Rather than rejecting the WHO distinction between IC and functional ability, this perspective argues that how IC is understood and assessed is increasingly shaped by the sociotechnical arrangements through which it is measured, classified, and acted upon. This relational understanding can be described as distributed IC: IC is enacted through relations among bodies, technologies, data systems, care environments, and institutional practices. Accordingly, equitable AI-supported aging care requires more than improving algorithmic accuracy. Gerontologists should be involved before datasets and thresholds are fixed; older adults should participate in design and evaluation; and algorithmic classifications should be explainable, reviewable, and contestable. From a new materialist gerontology perspective, the central question is not only whether AI systems work, but also how they shape what counts as capacity, whose needs become recognizable, and how care is organized in later life.
